# Early and Degressive Putamen Atrophy in Multiple Sclerosis

**DOI:** 10.3390/ijms161023195

**Published:** 2015-09-25

**Authors:** Julia Krämer, Sven G. Meuth, Jan-Gerd Tenberge, Patrick Schiffler, Heinz Wiendl, Michael Deppe

**Affiliations:** Department of Neurology, Westfälische Wilhelms University, Albert-Schweitzer-Campus 1, Gebäude A1, 48149 Münster, Germany; E-Mails: sven.meuth@ukmuenster.de (S.G.M.); jan-gerd.tenberge@ukmuenster.de (J.-G.T.); patrick.schiffler@ukmuenster.de (P.S.); heinz.wiendl@ukmuenster.de (H.W.)

**Keywords:** high-resolution structural MRI, FreeSurfer, volumetry, multiple sclerosis, early putamen atrophy, disease duration

## Abstract

Putamen atrophy and its long-term progress during disease course were recently shown in patients with multiple sclerosis (MS). Here we investigated retrospectively the time point of atrophy onset in patients with relapsing-remitting MS (RRMS). 68 patients with RRMS and 26 healthy controls (HC) were admitted to 3T MRI in a cross-sectional study. We quantitatively analyzed the putamen volume of individual patients in relation to disease duration by correcting for age and intracranial volume (ICV). Patient’s relative putamen volume (RPV), expressed in percent of ICV, was significantly reduced compared to HC. Based on the correlation between RPV and age, we computed the age-corrected RPV deviation (ΔRPV) from HC. Patients showed significantly negative ΔRPV. Interestingly, the age-corrected ΔRPV depended logarithmically on disease duration: Directly after first symptom manifestation, patients already showed a reduced RPV followed by a further degressive volumetric decline. This means that atrophy progression was stronger in the first than in later years of disease. Putamen atrophy starts directly after initial symptom manifestation or even years before, and progresses in a degressive manner. Due to its important role in neurological functions, early detection of putamen atrophy seems necessary. High-resolution structural MRI allows monitoring of disease course.

## 1. Introduction

For a long time, multiple sclerosis (MS) has been regarded as an inflammatory and demyelinating disease predominantly affecting the white matter (WM) of the human central nervous system [1,2,3,4,5,6,7,8]. At the beginning of the 21th century, grey matter (GM) pathology and axonal and neuronal degeneration emerged as important features of this “typical” WM disease [2,5]. By the application of new histopathological, immunohistochemical, and neuroimaging methods, the full extent of GM damage became increasingly clear [4]. Although the thalamus was examined most extensively in patients with MS [9], some studies also demonstrated the involvement of other subcortical structures such as the putamen [5]. By the application of different automated segmentation and volume estimation techniques, e.g., voxel-based morphometry [10,11], FreeSurfer [12], and FSL tools [13], putamen atrophy was demonstrated in untreated patients with clinically isolated syndrome (CIS) [14,15] and in patients with different types of MS [7,14,16,17,18,19,20,21]. Volume loss of the putamen was shown to be relevant for conversion to clinically definite MS and for disease progression [14,22]. Furthermore, recent studies could reveal progredient putamen atrophy in patients with CIS and different forms of MS [7,14,18,21]. Against this background, we aimed to investigate the time point of putamen atrophy onset in patients with relapsing-remitting MS (RRMS).

## 2. Results

### 2.1. No Difference of Age and Gender Distribution between Patients and Healthy Controls (HC)

We found no systematic difference of age between patients and healthy controls (RRMS: mean age = 36.50 years (y), SD = 9.77 y; HC: mean age = 37.0 y, SD = 13.77 y; *p* = 0.86). Furthermore, gender distribution was statistically comparable between patients and healthy controls (Pearson’s Chi-squared test: corrected *p* = 0.69).

### 2.2. Significant Reduced Absolute Putamen Volume (APV) in Patients Compared to HC

**Figure 1:**
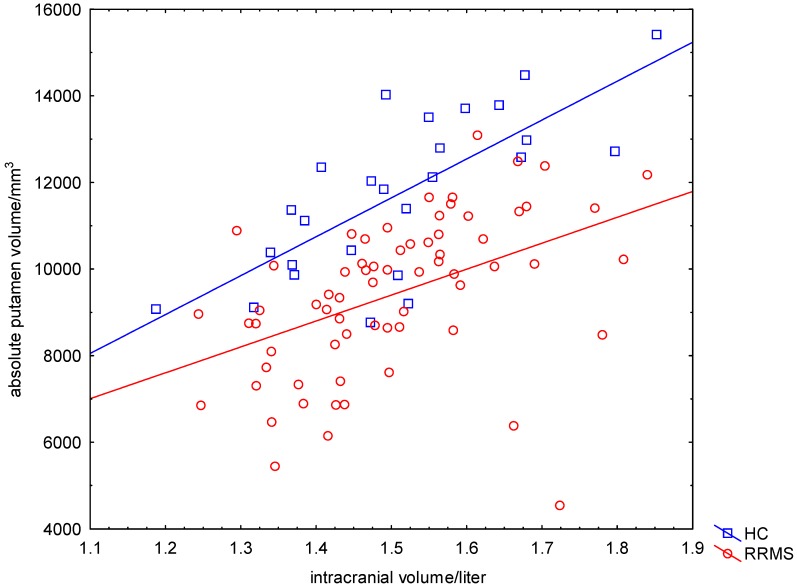
Significant correlation between APT and ICV. The absolute putamen volume (APV) in mm^3^ was strongly correlated with the intracranial volume (ICV) in liter in healthy controls (HC) and patients with relapsing-remitting multiple sclerosis (RRMS).

Patients’ and HCs’ ICVs were equivalently distributed (RRMS: mean ICV = 1.50 L, SD = 0.14 L; HC: mean ICV = 1.51 L, SD = 0.15 L; *p* = 0.84) (Figure 1). Patients with RRMS had on average a 20% lower APV compared to the group of HC (RRMS: mean APV = 9418 mm^3^, SD = 1794 mm^3^; HC: mean APV = 11,733 mm^3^, SD = 1849 mm^3^; *p* < 0.001) (Figure 1). The APV of male patients was larger than the APV of female patients (women: mean APV = 9062 mm^3^, SD = 1760 mm^3^; men: mean APV = 10,114 mm^3^, SD = 1685 mm^3^; *p* = 0.02). By employing a General Linear Model (GLM) (dependent variable APV, categorical predictor gender, continuous factor age), we found significant effects of age and gender on the APV (age: *p*_Age_ = 0.002; *F*_Age_ = 10.72; gender: *p*_gender_ = 0.007; *F*_gender_ = 7.62).

APV and ICV correlated significantly in all participants (RRMS: *p* < 0.001; HC: *p* < 0.001) (Figure 1). To avoid the confounding effects of ICV on APV, we computed the relative (percentage) putamen volume (RPV) (RPV = APV/ICV × 100%, Equation (7)).

### 2.3. Significant Reduced Relative Putamen Volume (RPV) in Patients Compared to HC

**Figure 2:**
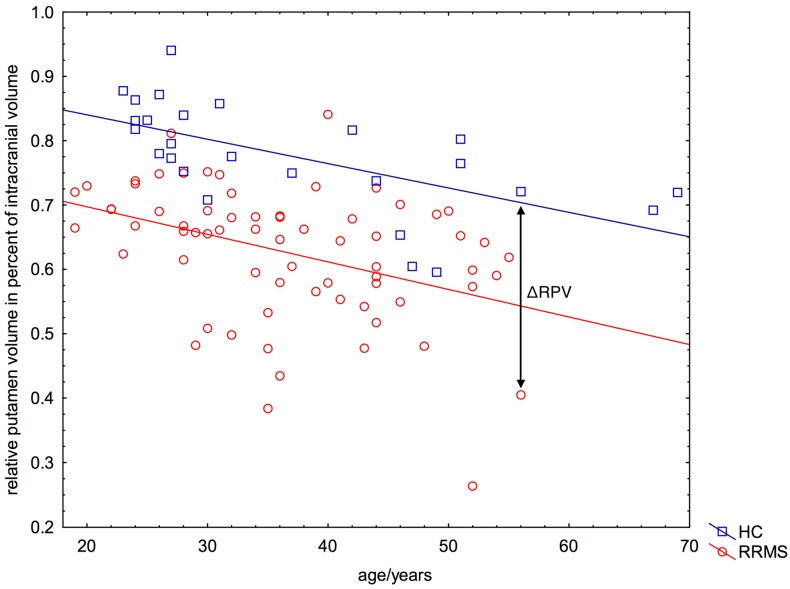
Significant correlation between relative putamen volume and participant’s age. Nearly every patient with relapsing-remitting multiple sclerosis (RRMS) (67/68) showed a lower RPV for a given age (=negative ΔRPV, see arrow), as expected from the relationship between RPV and age of healthy controls. The blue line represents the function RPV_HC_(Age) = 0.916 − 0.0038 × Age (Equation (1)).

RPV and the participants’ age correlated significantly in both the HC group (RPV_HC_(Age) = 0.916 − 0.0038 × Age; *p* < 0.001) and the RRMS group (RPV_MS_(Age) = 0.783 − 0.0043 × Age; *p* < 0.001) (Figure 2). Patients with RRMS had on average a 19% lower RPV compared to the group of HC (RRMS: mean RPV = 0.63%, SD = 0.10%; HC: mean RPV = 0.78%, SD = 0.8%; *p* < 0.001) (Figure 2). We found no influence of gender on the RPV of patients (women: mean RPV = 0.62%, SD = 0.11%; men: mean RPV = 0.64%, SD = 0.09%; *p* = 0.52).

### 2.4. Negative ΔRPV and ΔRPV% in Nearly All Patients

We used the RPV of HC (RPV_HC_) as the age-dependent predictor for the expected RPV of each patient with MS. The age-dependent predictor was calculated by linear regression (Figure 2, blue line) as

(1) $${\text{RPV}}_{\text{HC}}\left(\text{Age}\right)=0.916\;-\;0.0038\;\times \text{ Age}$$

The difference between a patient’s individual RPV (RPV_MS_) and the estimated RPV_HC_ was calculated as (Figure 2):

(2) $$\text{ΔRPV}={\text{RPV}}_{\text{MS}}\;-{\text{ RPV}}_{\text{HC}}$$

**Figure 3:**
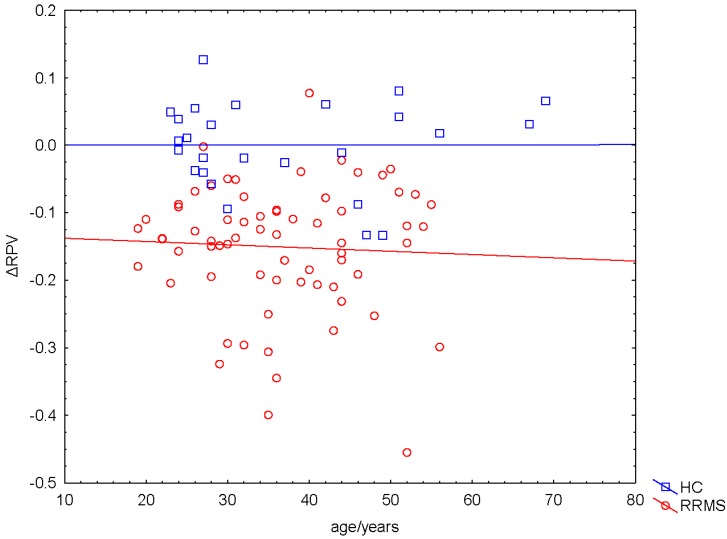
Independence of ∆RPV on participants’ age. ∆RPV did not depend on participants’ age (*p* = 0.64).

By employing a GLM (dependent variable ∆RPV, categorical predictor gender, continous factor age), we did not find significant effects of age and gender on ∆RPV (age: *p*_Age_ = 0.64; *F*_Age_ = 0.22; gender: *p*_Gender_ = 0.34; *F*_Gender_ = 0.94) (Figure 3). Therefore, we did not include patients’ gender and age as additional factors in the statistical analyses.

For each patient, we computed the percentage volumetric deviation of the patients’ putamen in relation to HC as:

(3) $$\text{ΔRPV\%}=\text{ΔRPV}/{\text{mean RPV}}_{\text{HC}}\times \;100\text{\%}$$

Nearly every patient (67/68) showed a lower RPV (=negative ΔRPV%) as expected from HC (RRMS: mean ΔRPV% = −19.4%, SD = 12.3%, lower quartile = −25.4%, median = −17.8%, upper quartile = −11.6%; HC: mean ΔRPV% = 0.0%, lower quartile = −4.8%, median = 1.1%, upper quartile = 6.3%; *t*-test on ΔRPV%: degrees of freedom = 92, *N*_RRMS_ = 68, *N*_HC_ = 26, *t* = −7.45, *p* < 0.001). ΔRPV% values of the HC group were symmetrically distributed around 0.0% because the regression function was estimated from this group.

### 2.5. Correlation between ΔRPV% and Volume of WM Lesions

ΔRPV% correlated significantly with the volume of WM lesions in T1w images (*R* = 0.66; *p* < 0.001) and FLAIR images (*R* = 0.67; *p* < 0.001).

### 2.6. Linear or Non-Linear Dependence of ΔRPV% on Disease Duration

According to Equation (9) we computed the parameters as *a* = −13.449, *b* = 5.116.

(4) $$\Delta \text{RPV\%}=5.116\;-\;13.449\;\times \;\log_{10}(\text{″disease duration″})$$

According to Equation (8) we computed the parameters as *a* = −0.083, *b* = −11.434

(5) ΔRPV%= −0.083 × ″disease duration″−11.434

**Figure 4:**
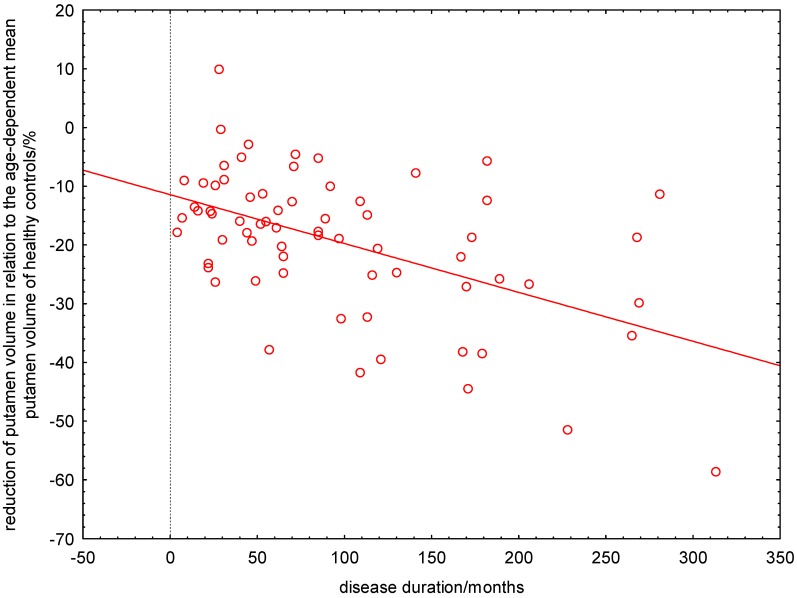
Linear estimation of age-corrected loss of putamen volume in relation to healthy controls *versus* the patients’ disease duration. At the manifestation of their first symptoms, patients had already a ΔRPV% of about 11% and lost every ten years further 10%.

**Figure 5:**
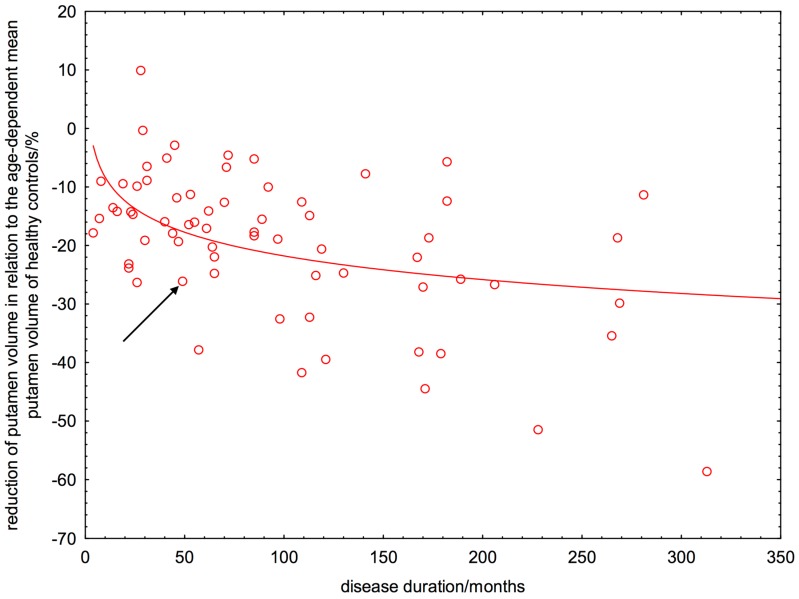
Logarithmic estimation of age-corrected loss of putamen volume in relation to healthy controls *versus* the patients’ disease duration. The arrow indicates a patient with a disease duration of 50 months who had a 26% lower putamen volume than expected from a healthy control with the same age as the patient.

The linear regression revealed that putamen atrophy is already present at manifestation of patients’ first symptoms and progresses continuously. At the manifestation of their first symptoms, patients had already a ΔRPV% of about 11% and lost every ten years further 10% (Figure 4). By using this linear model, the time point of putamen atrophy onset was estimated to minus 138 months. Systematic analysis of the residual variance (normal probability plot, sum of least squares) provided evidence that the non-linear model describes the data better than the linear estimation (Figure 5).

## 3. Discussion

### 3.1. Early and Degressive Putamen Atrophy in Patients with Relapsing-Remitting Multiple Sclerosis (RRMS)

The most important finding of our study was an early and degressively increasing putamen atrophy in patients with RRMS. Both the linear and the logarithmic model revealed that patients lose directly after first symptom manifestation or most likely even years before, putamen volume. The linear regression would estimate the time point of putamen atrophy onset to minus 138 months (Figure 4). This is in our view probably an overestimation. Moreover, the asymptotic behavior of the linear function is not compatible with a finite putamen volume. In contrast to the linear regression, the logarithmic model showed that the putamen atrophy started directly after manifestation of patients’ first symptoms and progresses in a degressive manner. This means that the atrophy progression is stronger in the first rather than the later years of the disease. In other words, a patient with a disease duration of ten years showed already a 17% reduced putamen volume compared to HC (Figure 5). The estimated MS-related further volume loss was only 3% in the second decade of the disease. By computing the relative percentage putamen volume (RPV), we avoided confounding effects of ICV and gender on putamen volumes. We demonstrated that aging did also not confound the volume loss seen in patients with RRMS, because we used the age-corrected ΔRPV as measure for putamen atrophy.

### 3.2. Interpretation of Results in Perspective of Previous Studies

Being aware of the important functions of the putamen, the consequences of this atrophy become clear. The putamen as part of the dorsal striatum and the basal ganglia plays a unique role in movement regulation, motor function, coordination, and cognition [15,18,23]. Previous studies demonstrated that iron deposition in the putamen correlates with EDSS and cognitive performance [23,24,25], and predicts disability progression [7,26]. By using FIRST as a segmentation tool, recent studies have already revealed progredient putamen atrophy in patients with RRMS and SPMS [14,18], without finding gender differences [21]. The putamen volume was, however, always computed over the whole group of patients [7,14,18,21] and not quantitatively in individual patients. Especially if the group consists of relapsing and progressive MS forms [7,18], this calculation could potentially imply—provided that neurodegenerative processes progress differently in patients with various types of MS—some limitations. Additionally, in the studies of Jacobsen, Dolezal, and Bergsland *et al.* no healthy subjects were included as controls [7,14,21]. However, progredient putamen volume loss cannot be declared as pathological in patients with MS without taking the normal age depended decline of the putamen of HC into account. In contrast to these studies, we computed the putamen volume for each individual patient in relation to disease duration. By using a logarithmic function to model the patients’ ΔRPV% in relation to disease duration, we could estimate the time point of atrophy onset and the time course of atrophy progression during the disease.

### 3.3. Limitations and Future Research

We employed a fully automated MR image analysis technique (a pipeline incorporating tools of the FreeSurfer software suite) for the volumetric analysis of cerebral structures (APV and ICV). An inherent problem with such an approach is that segmentation errors can result in falsified volumetric measures. However, the reliability and validity of FreeSurfer and its consistency with manual stereology, which is considered to represent the “gold standard” for volumetric MRI assessments [27,28,29,30], was recently demonstrated for the thalamic volume [31]. Therefore, we compared the FreeSurfer results with an independent toolset (FSL-FIRST), which provided highly comparable results (*R* = 0.92). The present study was performed on a cross-sectional basis. Longitudinal studies are necessary to demonstrate individual progredient putamen atrophy and to investigate whether early volumetric alterations can serve as predictors of future disease course and as surrogate markers for treatment effects. Another limitation of our study is that we disregarded inter-individual differences concerning disease-modifying therapy at the moment of MRI examination. For further studies, it would be interesting to analyze the neurological and neuropsychological consequences of putamen volume loss. In addition, the difference of onset and development of putamen atrophy of our examined patients in dependence of the presence of oligoclonal immunoglobulin G bands in the cerebrospinal fluid should be another objective. Recently, Ferreira *et al.* could demonstrate that patients with MS lacking oligoclonal immunoglobulin G bands in the cerebrospinal fluid have less global and regional brain atrophy, also in the basal ganglia [32]. We are currently investigating whether certain subcortical structures are excluded from neurodegenerative processes and whether atrophy onset and development differ among subcortical structures in MS. Because the thalamus has widespread reciprocal connections with subcortical structures as the putamen, it would be interesting to analyze the relationship of volumetric and microstructural alterations of the thalamus and the putamen in patients with MS.

## 4. Experimental Section

### 4.1. Subjects and Their Main Clinical and Imaging Features

We recruited 68 consecutive patients (45 women, 23 men) in our clinic with relapsing-remitting MS (RRMS) diagnosed according to the revised McDonald criteria [33]. Additionally, 26 age-matched subjects (19 women, 7 men) without any history of neurological and psychiatric diseases were included as healthy controls (HC). The latter were recruited by announcements in local newspapers. Table 1 provides descriptive demographic details of all examined 94 participants. The following exclusion criteria were applied for all patients: any pre-existing medical condition known to be associated with brain pathology, pregnancy, previous or current addiction to substances, relapses or systemic therapy with steroids (intravenous, intrathecal, or oral) within the month before the MRI, history of additional neurological or psychiatric disorders. At the moment of MRI examination, all patients were neurologically examined. Written informed consent to participate in this study was obtained from all subjects. The participants were informed that the MRI examination could reveal potential medically significant findings and were also given the option to request notification in the event of such findings. The study was approved by the ethics committee of the University of Münster and the Physicians’ Chamber of Westphalia-Lippe (Ärztekammer Westfalen-Lippe, 2010-378-b-S, 21 October 2010).

**Table 1:** Descriptive statistics about all examined participants. We found no systematic age difference between patients and healthy controls (*p* = 0.86).

Patients	Mean	Median	Min.	Max.	Lower Quartile	Upper Quartile	Standard Deviation
Age/years	36.5	36.0	19.0	56.0	29.0	44.0	9.8
White matter volume/L	0.46	0.45	0.31	0.63	0.41	0.49	0.06
Grey matter volume/L	0.68	0.68	0.54	0.81	0.62	0.71	0.06
Intracranial volume/L	1.5	1.5	1.2	1.8	1.4	1.6	0.1
Disease duration/months	96.1	70.5	4.0	313.0	35.5	135.5	76.5
Expanded Disability Status Scale	2.1	2.0	0.0	6.0	1.5	2.5	1.3
White matter lesion volume in Fluid-attenuated inversion recovery images/mL	9.6	4.9	0.0	100.6	1.4	11.7	15.1
White matter lesion volume in T1-weighted images/mL	4.0	2.7	0.4	30.8	1.6	4.3	5.0
**Healthy Controls**							
Age/years	37.0	30.5	23.0	69.0	26.0	47.0	13.8
White matter volume/L	0.52	0.52	0.41	0.70	0.47	0.57	0.07
Grey matter volume/L	0.73	0.73	0.54	0.86	0.68	0.80	0.09
Intracranial volume/L	1.5	1.5	1.2	1.9	1.4	1.6	0.2

### 4.2. Magnetic Resonance Imaging

All participants were scanned using the same 3T Siemens TIM Trio MRI scanner and a 12-channel (matrix) head coil (Siemens AG, Erlangen, Germany). Employing the same MRI parameters and protocols, we obtained native isotropic 3D MPRAGE T1-weighted (T1w) images (field of view (FOV) 256 × 256 mm^2^, slice thickness 1.0 mm, matrix 256 × 256, no gap, repetition time (TR) 2000 ms, echo time (TE) 2.52 ms, generalized autocalibrating partially parallel acquisition (GRAPPA) factor) for all subjects. The following MRI sequences were only applied to the patients with RRMS: an axial turbo spin-echo (TSE) Fluid-attenuated inversion recovery (FLAIR) (44 slices, FOV 250 × 250 mm^2^, slice thickness 3.0 mm, matrix 256 × 256, TR 9200 ms, TE 88 ms, no gap, slice order interlaced, TI (inversion time) 2300 ms, flip angle 150°), a sagittal TSE FLAIR (32 slices, FOV 240 × 240 mm^2^, slice thickness 3.0 mm, matrix 256 × 256, no gap, slice order interlaced), and a 3D MPRAGE T1w after intravenous gadolinium-DTPA (diethylene triamine penta-acetic acid) injection (0.1 mmol/kg). Foam paddings minimized head motion.

### 4.3. Automated Volumetric Analysis of Cerebral Structures

All 3D MPRAGE images were corrected for contrast and intensity inhomogeneities to reduce segmentation errors using an in-house software (Eval 3.0). Total WM and GM volume, putamen and intracranial volume (ICV), and volume of hypointense WM lesions in T1w images were obtained from FreeSurfer (Version 5.1; [12]). For details of this method see [34,35,36,37]. Additionally, we calculated the putamen volume from FSL-FIRST (Integrated Registration and Segmentation Tool, Oxford University, Oxford, UK), which is incorporated into the FSL software (Version 5.0; [13]). For details of this method see [38]. By the application of these two alternative techniques, we achieved quite similar results by Pearson product-moment correlation (*R* = 0.92). Due to the congruent results between FSL-FIRST and FreeSurfer, we decided to present only the obtained by FreeSurfer. Volume of hyperintense WM lesions in FLAIR images was obtained from LST (Lesion Segmentation Tool) (Version 1.2.3; [39]) [40]. We calculated the absolute putamen volume (APV) (unit: mm³) for all participants as

(6) $$\text{APV}=\text{right putamen volume }+\text{ left putamen volume }\left[{\text{mm}}^{3}\right]$$

From the APV and the absolute ICV (unit: mm³) we calculated the relative (percentage) putamen volume as

(7) RPV=APV/ICV× 100%

Disease duration was defined as time between the manifestation of patient’s first symptoms and the date of MRI examination.

### 4.4. Statistical Analysis

Data were analyzed using STATISTICA™ version 10 (StatSoft (Europe) GmbH, Hamburg, HH, Germany). By performing the Pearson’s Chi-squared test, we analyzed the gender distribution between patients and healthy controls. Differences of age, ICV, APV, RPV, and ΔRPV% (the percentage deviation of the patients’ putamen volume in relation to HC) between patients and HC were tested by two-sided *t*-tests. Differences were considered as statistically significant if *p* ≤ 0.05. By linear univariate regression analyses we calculated correlation coefficients of APV and ICV, of RPV and participant’s age, of ΔRPV and participant’s age, and additionally of ΔRPV% and volume of WM lesions in T1w and FLAIR images. To examine the relation between ΔRPV% and the patient’s disease duration, we used the linear function

(8) ΔRPV% = a + b × ″disease duration″

and the logarithmic function (a and b estimated by lest squares fitting)

(9) $$\text{ΔRPV\% }=\text{ a }\times \;\log_{10}(\text{″disease duration″})+\text{ b}$$

## 5. Conclusions

Putamen atrophy occurs directly after manifestation of first symptoms or most likely even years before—at a pre-diagnosis time point—and progresses in a degressive manner in patients with RRMS, far beyond normal aging. Considering the important role of the putamen in movement regulation, motor function, coordination, and cognition, early detection of putamen volume loss in patients with MS seems necessary. High-resolution structural MRI offers the possibility to determine precisely pathological volumetric alterations of grey matter structures in patients with MS and therefore seems to be a useful tool to monitor disease course and therapy response.

## Abbreviations

APV = absolute putamen volume; CIS = clinically isolated syndrome; EDSS = Expanded Disability Status Scale; FIRST = FMRIB Integrated Registration and Segmentation Tool; FLAIR = Fluid-attenuated inversion revovery; FOV = field of view; GLM = General Linear Model; GM = grey matter; HC = healthy controls; ICV = intracranial volume; LST = Lesion Segmentation Tool; MRI = magnetic resonance imaging; MS = multiple sclerosis; RRMS = relapsing-remitting multiple sclerosis; RPV = relative putamen volume; ΔRPV = difference between the age-dependent RPV of a patient and the expected RPV based on age-dependent values of healthy controls; ΔRPV% = percentage deviation of patients’ putamen volume in relation to healthy controls; SD = standard deviation; SPMS = secondary progressive MS; TI = inversion time; TR = repetition time; TSE = turbo spin-echo; T1w = T1-weighted; WM = white matter; y = years.
